# Smoking

**Published:** 1869-10

**Authors:** 


### SMOKING.

One of the most stupid, contemptible, and filthy sights in our eye, is to see a grown-up man stuck behind a cigar or dirtier pipe, spending whole hours in drawing smoke into his mouth and then puffing it out, sleepily gazing up at its curling wreaths as if there was something entrancing in the sight. When we take into account the useless expense, the bootless trouble, and the beastly scattering about of the noisome saliva incident to the habit, the wonder is that any man of intelligence should cultivate the despicable slavery, such a relentless despotism; that it is so, no one can deny, for if an habitual smoker has not his cigar at the accustomed time, he is literally miserable, and by his peevishness, fretfulness, and irritability makes all around him as uncomfortable as himself. Suppose he is to go on a journey, the overshadowing thought of his heart, his first, his middle and his last concern is that he does not leave without a full supply of his dearly-beloved cigar; and if by any means it should be left or lost, he is one of the most miserable of men. Shame on the intelligence that cannot summon courage to break such a chain on the instant into ten thousand atoms. A tobacco smoker without his pipe is no man at all; he is not himself, and the miserable habit grows on him day by day, until at length, he is only human or humane when he has a pipe in his lips. He can't drive his horse without a cigar in his mouth; he can't make a trade unless he is smoking. What cares he for the convenience and comfort of others; what for the respect due to ladies? Why, rather than not smoke, he would drive a dozen people from an omnibus, or half a hundred from a railroad car! There he goes strutting along the street, brushing by ladies, and allowing the filthy fumes to dash against their faces; fumes made more filthy from having gathered worse odors from his rotten teeth and the slimy saliva which is plastered over his inner cheeks. The next thing is to spit on the side-walk and have it wiped up by the dress of some unfortunate passer by. But what cares a smoker for considerations like these? Only give him a smoke, and all else is as nothing to him; so supreme is he in his selfishness, that respect, gallantry, good feeling, all are lost.

It so happens that the two men who fill the most important stations in their line in the civilized world are helpless slaves to the miserable practice. The Emperor of the French was dying of the habit of smoking seventeen cigars a day, and was compelled by imperative medical authority to cut it down to seven; and about the same time our own President had to curtail his indulgence in the same direction, or imperil a nation's interests. Reader, are you a smoker? Then you are literally and unmistakably a fool. There is no use in playing the courtier here, as long as you are a slave to the pipe and to the cigar; for you are a voluntary slave to a useless, expensive, filthy, and hurtful habit. Is it not a folly to prefer slavery to freedom, and that a disgusting slavery?

